# Abnormal Somatosensory Behaviors Associated With a Gain-of-Function Mutation in TRPV3 Channels

**DOI:** 10.3389/fnmol.2021.790435

**Published:** 2022-01-04

**Authors:** Mahar Fatima, Hannah Slade, Lorraine Horwitz, Angela Shi, Jingyi Liu, Delaney McKinstry, Troy Villani, Haoxing Xu, Bo Duan

**Affiliations:** Department of Molecular, Cellular, and Developmental Biology, University of Michigan, Ann Arbor, MI, United States

**Keywords:** TRPV3, somatosensation, gain-of-function, G573S, epidermal hypoinnervation

## Abstract

Thermosensitive transient receptor potential V3 (TRPV3) is a polymodal receptor implicated in nociceptive, thermoceptive, pruritoceptive, and inflammatory pathways. Reports focused on understanding the role of TRPV3 in thermoception or nociception are not conclusive. Previous studies also show that aberrant hyperactivity of TRPV3 channels results in spontaneous itch and dermatitis-like symptoms, but the resultant behavior is highly dependent on the background of the animal and the skin microbiome. To determine the function of hyperactive TRPV3 channels in somatosensory sensations, we tested different somatosensory behaviors using a genetic mouse model that carries a gain-of-function point mutation *G573S* in the *Trpv3* gene (*Trpv3*^*G*573*S*^). Here we report that *Trpv3*^*G*573*S*^ mutants show reduced perception of cold, acetone-induced cooling, punctate, and sharp mechanical pain. By contrast, locomotion, noxious heat, touch, and mechanical itch are unaffected in *Trpv3*^*G*573*S*^ mice. We fail to observe any spontaneous itch responses and/or dermatitis in *Trpv3*^*G*573*S*^ mutants under specific pathogen (*Staphylococcus aureus*)-free conditions. However, we find that the scratching events in response to various pruritogens are dramatically decreased in *Trpv3*^*G*573*S*^ mice in comparison to wild-type littermates. Interestingly, we observe sensory hypoinnervation of the epidermis in *Trpv3*^*G*573*S*^ mutants, which might contribute to the deficits in acute mechanical pain, cool, cold, and itch sensations.

## Introduction

The transient receptor potential (TRP) superfamily of cation channels acts as molecular sensors for chemical, thermal, and mechanical stimuli to evoke various sensory sensations such as heat, cold, pain, and itch. TRPV3 is a TRP family member which is a warm temperature-responsive channel with reported temperature thresholds of activation ranging from 31 to 39°C (Peier et al., 2002; Smith et al., 2002; Xu et al., 2002). TRPV3 is highly expressed in the epidermal keratinocytes (Peier et al., 2002; Xu et al., 2002) and is involved in skin barrier function, hair growth, and skin inflammation (Cheng et al., 2010; Luo and Hu, 2014). TRPV3 channels are also implicated in sensing temperatures and pain in rodents, but those results remain controversial. Genetic deletion of *Trpv3* produces selective deficits in responses to innocuous and noxious heat (Moqrich et al., 2005), but the contribution of TRPV3 channels in thermal sensations depends on the genetic background (Huang et al., 2011; Miyamoto et al., 2011). *Trpv3* deletion on the 129S6 background resulted in a preference for cooler temperatures (22–32°C), whereas *Trpv3* null mice on the C57BL6 background display no obvious alterations in thermal preference (Huang et al., 2011). These results suggest that TRPV3 channels may have limited and strain-dependent contributions to thermosensation. In addition to temperature sensations, TRPV3 channels also mediate chemical-induced nocifensive behaviors. Activation of TRPV3 channels with the agonist farnesyl pyrophosphate (FPP) elicits rapid pain-related responses in mice under inflammatory conditions (Bang et al., 2010), and the FPP-evoked nocifensive response is significantly reduced by blocking TRPV3 channels (Bang et al., 2011, 2012). However, another study has demonstrated that TRPV3 null mice on both C57BL6 and 129S1/Svlm background exhibit no change in acute heat pain or inflammatory heat hyperalgesia (Huang et al., 2011). Moreover, non-selective agonists of TRPV3, carvacrol and thymol, induce analgesia and are anti-inflammatory (Cavalcante Melo et al., 2012; Guimaraes et al., 2012; Nagoor Meeran et al., 2017). These results argue against the pro-nociceptive role for native TRPV3 channels.

*Nh* (non-hair) mutation was reported for the first time in DS mice when this spontaneous mutation was found to produce a hairlessness phenotype accompanied by the development of allergic and pruritic dermatitis in the presence of a specific pathogen, namely *Staphylococcus aureus* (Haraguchi et al., 1997). By contrast, in the absence of the pathogen *S. aureus*, the *Nh* mutant mice do not develop spontaneous dermatitis and scratching (Haraguchi et al., 1997; Asakawa et al., 2006; Imura et al., 2009). Moreover, altering the background of *Nh* mutant from DS to NC/Nga, the gain-of-function *Nh* mutation fails to produce any spontaneous scratching or dermatitis even in the presence of *S. aureus* (Imura et al., 2009). The *Nh* is shown to be a point mutation that changes glycine to serine at position 573 in the *Trpv3* gene (Asakawa et al., 2006). This point mutation in the *Trpv3* gene renders TRPV3 channels constitutively open and hence hyperactive (Xiao et al., 2008). In humans, gain-of-function genetic mutations of *TRPV3* cause Olmsted syndrome, which is clinically characterized by diffuse palmoplantar keratoderma, alopecia, and skin inflammation (Poulin et al., 1984; Lucker and Steijlen, 1994; Nofal et al., 2010; Lai-Cheong et al., 2012). However, around 52% of Olmsted cases show painful palmoplantar keratoderma, and about 22% of patients report pruritus (Mevorah et al., 2005; Tao et al., 2008). These findings suggest that itch sensitization in DS-*Nh* mice or patients with Olmsted syndrome might not be directly caused by the hyperactivation of TRPV3 channels. Recent studies focus on the function of TRPV3 channels in acute chemical itch transmission (Sun et al., 2020; Zhao et al., 2020; Han et al., 2021). However, none of the studies have provided conclusive evidence of how TRPV3 is involved in itch sensation.

In this study, we tested the role of hyperactive G573S mutation in the *Trpv3* gene in various somatosensory behaviors such as acute thermal, pain, and itch sensations to evaluate the role of TRPV3 in regulating various somatosensory modalities. We found that acute chemical itch, mechanical pain, and cool/cold sensations were largely attenuated in *Trpv3*^*G*573*S*^ mutant mice on the 129S1/Svlm genetic background, while locomotion, noxious heat pain, touch, and mechanical itch sensations remained unaffected. We also observed a drastic reduction of epidermal sensory innervation in *Trpv3*^*G*573*S*^ mice, which might cause the notable deficits that we observed in the gain-of-function *Trpv3*^*G*573*S*^ animals.

## Materials and Methods

### Animal Behavior

All animal experiments were performed in accordance with protocols approved by the Institutional Animal Care and Use Committee at the University of Michigan. *Trpv3*^G573S^ mutant animals were obtained from Dr. Takeshi Yoshioka, Shionogi Discovery Research Laboratories, Japan. We re-derived the *Trpv3*^G573S^ mutants using 129S1/SvlmJ eggs; the animals obtained were then backcrossed to 129S1/SvlmJ background for six-eight generations. These backcrossed 129S1/SvlmJ *Trpv3*^G573S^ mutants and control littermates were used for all the experiments. Animals were housed at room temperature with *ad libitum* access to standard lab mouse pellet food and water. A 12 h light/12 h dark cycle was maintained in the animal housing area. Littermates of both sexes were used in the experiments. Prior to the behavioral evaluation, animals were habituated in the testing apparatus for 20–30 min for three to 5 days.

#### Acute Chemical Itch

Acute itch tests were performed as described previously (Liu et al., 2010; Fatima et al., 2019). 100 μg of Compound 48/80 (MilliporeSigma, St. Louis, MO, United States), 50 μg of chloroquine (MilliporeSigma, St. Louis, MO, United States), 50 μg of PAR2 agonist SLIGRL-NH2 (Bachem, Switzerland), or 100 μg of serotonin agonist α-Me-5-HT (MilliporeSigma, St. Louis, MO, United States) in 50 μl of sterile saline was injected in the nape of the animal. The behavior was recorded for an hour and analyzed later. The scratching responses were counted in a period of 45 min post pruritogen delivery.

#### Dynamic Touch Test

Dynamic touch was tested by stroking a soft brush in the direction of the heel to toe for three times, delivering each stimulus after a 10-s interval. No response was scored as 0, whereas when the animal moved away, and/or a swift paw lifting was scored as 1. Cumulative response in three trials was represented as touch score.

#### Pinprick Test

Animals were placed in a plexiglass chamber on an elevated wire grid platform that exposed the plantar surface to be stimulated with a sharp pin. The plantar surface was poked with the pin without penetrating the pin into the skin. The pin was pricked 10 times, repeating the stimulus every 1–2 min on different areas of the plantar hind paw surface. The number of paw withdrawals in response to the stimulus was recorded.

#### Von Frey Test

Mice were placed in a plexiglass chamber on an elevated wire grid platform. The glabrous surface of the paw was probed with a set of calibrated von Frey filament (0.016–2 g). The paw withdrawal response was captured, and scores were calculated following Dixon’s up-down method (Chaplan et al., 1994).

#### Acetone-Induced Cooling Test

Animals were placed on an elevated mesh chamber. A drop of acetone was deposited on the glabrous paw of the animal using a syringe mounted with a plastic tubing. The stimulus was applied once every 30 s, twice per paw, and alternating between paws. The test was repeated four times in total and averaged to represent a score. All of the behavioral responses were videotaped and analyzed later. To identify nocifensive and cool-induced aversive behaviors but not touch, hind paw flinch was scored as 1; a single lick was scored as a 2; multiple licks was scored as 3, guarding, vocalization, and/or escape behaviors were scored as 4.

#### Hot Plate and Cold Plate Test

Animals were placed on a hot/cold plate (IITC, Woodland Hills, CA, United States) maintained at either 0, 46, 50, or 54°C, and the response time for a hind paw or forepaw lick was measured. To avoid nocifensive injury, a cutoff time to remove the animal from the plate was set at 120, 60, 30, and 20 s for assays performed at 0, 46, 50, and 54°C, respectively. For the cold plate assay, the latency to lick the forepaw was recorded. For the hot plate assay, the latency to lick the hindpaw was recorded. All the behaviors were videotaped and analyzed later.

#### Rota-Rod Assay

To test sensorimotor coordination, animals were placed on accelerating rotarod (IITC, Woodland Hills, CA, United States), and the time taken by the animal to fall from the rod was recorded. The test was repeated three times and averaged to represent a score.

### Immunostaining

#### DRG Immunostaining

Animals were transcardially perfused with 4% paraformaldehyde. Lumbar DRG L4 and L5 were dissected, cryoprotected in 20% sucrose, mounted in OCT media, and sectioned at a thickness of 12 μm. Slides were washed with 1× PBS thrice (2 min for each wash). The sections were blocked with 0.2% Triton X-100 in 10% NGS for 30 min. Sections were washed with 1× PBS twice (2 min for each wash). Sections were incubated with primary antibodies diluted in a blocking buffer overnight in a humid chamber (Anti-CGRP, Peninsula labs, San Carlos, CA, United States). After the incubation of primary antibodies, sections were washed with 1× PBS three times (2 min for each wash) and then incubated the sections with secondary antibodies (1:1000) and Alexa Fluor 488-conjugated Isolectin GS-IB4 (Thermo Fisher Scientific, Waltham, MA, United States) in a humid chamber. Finally, the sections were washed with 1× PBS five times (5 min for each wash). For counting, L3 and L4 DRG pairs from three different animals were counted.

#### Skin Immunostaining

Glabrous skin from the hind paw was removed and fixed in Zamboni’s buffer for 2 h and then transferred to sucrose solution for 4 h. Tissues were mounted in OCT and sectioned at a thickness of 30 μm. Free-floating sections were then incubated with primary antibodies against β-tubulin (R&D Systems, Minneapolis, MN, United States). Sections were washed three times with 1× PBS (5 min for each wash) followed by incubation with appropriate secondary antibodies. Again, sections were washed five times with 1× PBS (5 min for each wash). Sections were mounted and imaged using Leica Sp8 confocal microscope. The representative images were a composite of captured *z*-stacks. Nerve endings from 4–10 images captured for each group were counted and represented as an average. To quantify the nerve density in the epidermis in these images, we followed an assessment method described previously (Ebenezer et al., 2007). When the branches of nerve terminals sprout out before crossing the basement membrane of the epidermis, each branch is considered an individual unit, nerve terminals that branched after crossing the basement membrane were considered as one single fiber, each nerve fragment located in the epidermis was counted as one. Branches of the epidermal nerve fragments were not counted. Nerve terminals that truncated in the dermis and did not cross the basement membrane were also not counted. The schematic representation for the skin epidermis was created using BioRender.

For TRPV1 immunostaining, 30 μm thick floating sections were probed with TRPV1 (Thermo Fisher Scientific, Waltham, MA, United States) overnight. The antibody was diluted in 1XPBST and 10% NGS. The next day, samples were washed with 1× PBST three times every 5 min. Samples were then incubated with secondary-conjugated with biotin for overnight incubation and washed every 5 min with 1× PBST. The sections were then treated with Streptavidin-HRP overnight and washed the next day. The sections were then incubated with 0.3% H_2_O_2_ for 15 min and then washed for another half an hour every 5 min. The samples were then incubated with TSA-Cy5 overnight and washed five times every 5 min. The samples were then mounted on the slides, and images were captured using Leica Sp8. To calculate the TRPV1 fluorescence intensity, we used ImageJ to evaluate the integrated density of five random regions of interest (ROIs) (∼200 μm^2^) from each section to sample the fluorescence intensity. The integrated intensities were averaged to represent the cumulative integrated density of fluorescence.

### Statistics

Data shown is represented as mean ± SEM. Statistical analysis was performed using the Prism (GraphPad). Statistical significance between different experimental groups was calculated using unpaired Student’s *t*-test; *p* < 0.05 was considered statistically significant.

## Results

### Aberrant Thickening of the Skin and Decreased Numbers of Epidermal Nerve Terminals of *Trpv3*^*G*573*S*^ Mice

**FIGURE 1:**
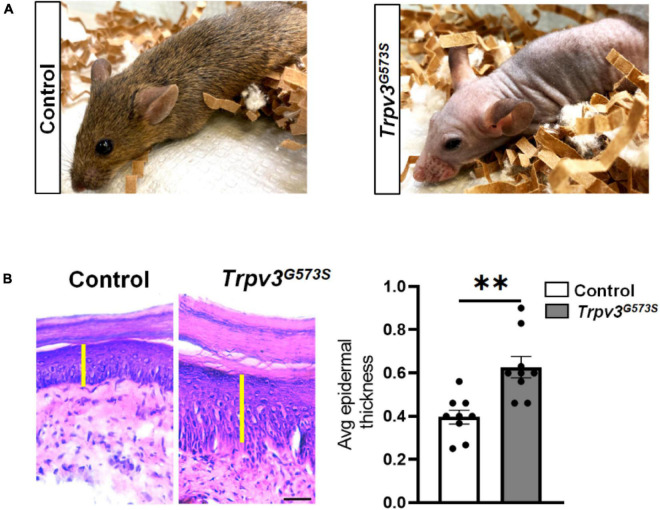
*Trpv3*^*G*573*S*^ mutant mice is hairless and has thicker epidermis. **(A)** Panel shows photographs of 8-weeks old control littermate and *Trpv3*^*G*573*S*^ mutant. **(B)** H&E-stained skin sections from the control and *Trpv3*^*G*573*S*^ mutant mice (scale 100 μm). Yellow bar indicates the epidermal layer of the skin which is evidently thicker in the mutant mice as compared to the control. Right panel shows bar graph represents the quantification for thickness of the epidermis in the control and mutant mice (*n* = 9 in each group), ^∗∗^*p* < 0.01, Student’s unpaired *t*-test.

**FIGURE 2:**
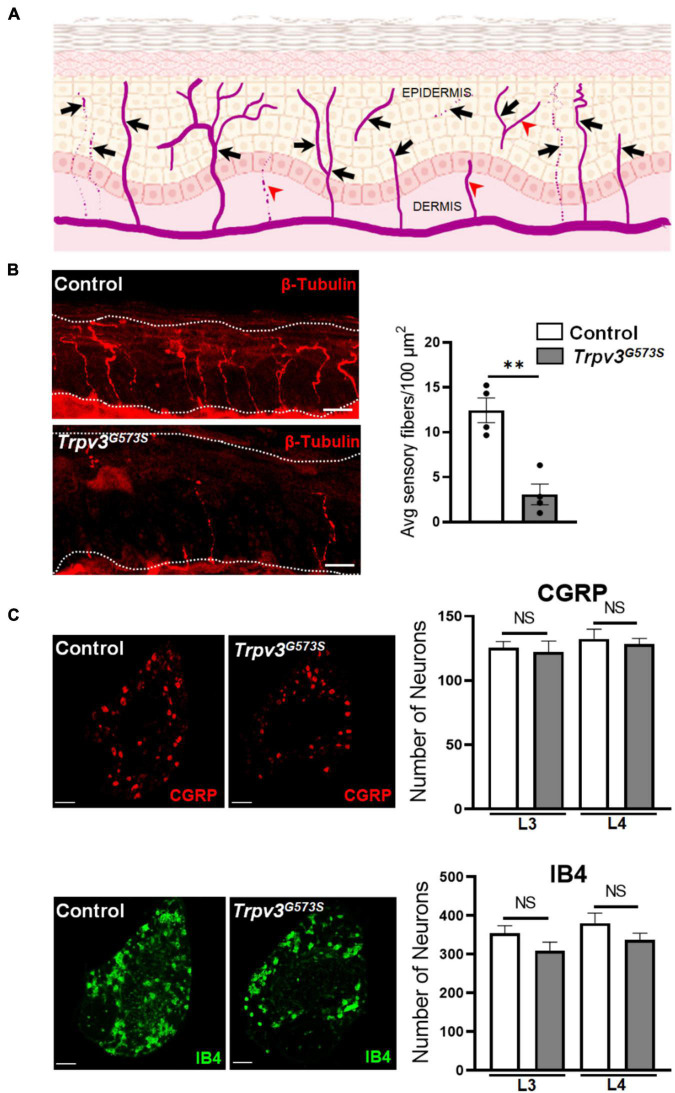
Analysis of nerve endings in the epidermis and number of C-subtype DRG neurons in *Trpv3*^*G*573*S*^ mutant mice and control littermates. **(A)** Schematic showing the template used for the analysis of epidermal innervation pattern. The arrows show the nerve terminals that were considered as a single unit of sensory innervation and fibers marked by arrowheads were not counted in the analysis. **(B)** Sensory endings in the epidermis in *Trpv3*^*G*573*S*^ mutant and control. Skin sections showing epidermal nerve terminals stained with β-tubulin in control and *Trpv3*^*G*573*S*^ mutant (scale 50 μm). Right panel shows bar graph representing the quantification of average number of epidermal sensory terminals in control and *Trpv3*^*G*573*S*^ mutant in 100 μm^2^ of skin area of the glabrous hind paw (*n* = 4 in each group). White dashed line in the images indicate the extent of epidermis. **(C)** Immunochemistry images showing the labeling for peptidergic CGRP-positive neurons and non-peptidergic IB4-positive neurons. Bar graph represents the numbers of CGRP- and IB4-positive neurons that were counted in L3 and L4 dorsal root ganglion in the *Trpv3* G573S mutant and control littermates (*n* = 3 in each group). Scale 100 μm. NS, no significant difference, ^∗∗^*p* < 0.01, Student’s unpaired *t*-test.

The TRPV3 channel has been documented to contribute to skin and hair architecture. Mice carrying *Trpv3*^*G*573*S*^ mutation showed abnormal hair coat with dramatically reduced hair length and diameter resulting in the phenotype of nearly naked skin (Figure 1A). The atypical activity of TRPV3 channels leads to greater epidermal thickness in the layer of stratum corneum (hyperkeratosis), granular layer (hypergranulosis), stratum spinosum, and stratum basale (Figure 1B), consistent with previous studies (Asakawa et al., 2006; Yoshioka et al., 2009). To understand the pattern of innervation of the *Trpv3*^*G*573*S*^ mutant, we tested the skin sections for β-tubulin, a pan-neuronal marker that can also label the sensory nerve terminals in the skin. Following a defined assessment method for counting epidermal nerve fibers (Ebenezer et al., 2007), we evaluated the number of all the nerve terminals in the epidermis (Figure 2A). Interestingly, we observed decreased innervation in the epidermis of the *Trpv3*^*G*573*S*^ mutants in comparison to control (Figure 2B). The unmyelinated polymodal C-afferents constitute a major population that innervates the skin epidermis and mediates nociceptive, pruriceptive, and temperature-sensitive somatic responses. The C-fibers can majorly be marked by the expression of Calcitonin Gene-Related Peptide (CGRP) and Isolectin B4 (IB4). To apprehend whether the decreased innervation in *Trpv3*^*G*573*S*^ mutants is due to the loss of small diameter C-subtype sensory neurons or because of mere loss of epidermal C-afferents, we counted the numbers of peptidergic (marked by CGRP) and non-peptidergic neurons (marked by IB4) in *Trpv3*^*G*573*S*^ mutants. The quantified data from L3 and L4 DRG showed no significant differences in the number of CGRP- and IB4-positive neurons in *Trpv3*^*G*573*S*^ mutant animals in comparison to control (Figure 2C).

### Thermal, Touch, and Pain Responses in *Trpv3*^*G*573*S*^ Mice

To inquire how such a dramatic increase in skin thickness and decreased density of epidermal nerve innervation in these *Trpv3*^*G*573*S*^ mutants can affect different somatosensations, we evaluated these mutants for various somatosensory behaviors.

**FIGURE 3:**
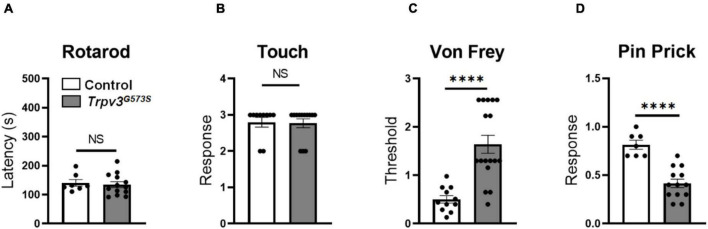
Evaluation of *Trpv3*^*G*573*S*^ mutants for sensory-motor coordination, dynamic touch, and mechanical stimuli. **(A,B)** Bar graph showing no significant difference in sensorimotor coordination or sensation of touch in mutant and control littermates (Rotarod: *n* = 11 for control, *n* = 17 for mutant, Touch: *n* = 10 for control, *n* = 13 for mutant). **(C)** Bar graph showing evaluation of paw withdrawal threshold calculated in response to mechanically delivered force through von Frey fibers in *Trpv3*^*G*573*S*^ mutants compared to control littermates (*n* = 11 for control, *n* = 17 for mutant). **(D)** Bar graph representing withdrawal response to pin prick in *Trpv3*^*G*573*S*^ mutant in comparison to control littermates (*n* = 11 for control, *n* = 13 for mutant). NS, no significant difference, ^∗∗∗∗^*p* < 0.0001, Student’s unpaired *t*-test.

Initially, we tested sensory-motor coordination in the *Trpv3*^G573S^ mutant and found it to be unchanged in comparison to control littermates (Figure 3A). Also, we tested the sensation of touch in these mutant animals. A soft brush was stroked from heel to toe direction three times with an interval of 10 s between each trial, and we found no evident difference between control and *Trpv3*^G573S^ mutant (Figure 3B). Hence, the hyperactivity of the TRPV3 channel does not have any adverse effect on sensory-motor coordination and touch sensation.

The physiological contribution of TRPV3 in pain signaling is still not completely understood. Using the gain-of-function TRPV3 mice model, we tested mechanical pain behavior in *Trpv3*^G573S^ mutants using the von Frey and pin-prick assays. *Trpv3*^G573S^ mutants showed an increased mechanical threshold for punctate mechanical stimulation determined by the von Frey assay (Figure 3C). Additionally, we noted a trend of decreased response to a sharp pain in the mutant animals in comparison to control littermates in the pin-prick test (Figure 3D). Hence, we observed that the hyperactive TRPV3 channel could lead to desensitization to sharp and punctate mechanical pain.

**FIGURE 4:**
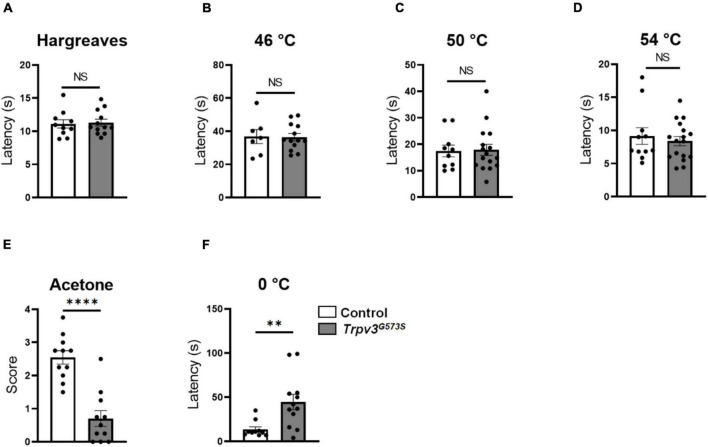
Response of *Trpv3*^*G*573*S*^ mutant to heat, cool and cold. **(A)** Bar graph showing latency of withdrawal to radiant heat representing noxious heat threshold in *Trpv3*^*G*573*S*^ mutants and control littermates (*n* = 10 for control, *n* = 12 for mutant). **(B–D)** Bar graphs represent latency to lick in response to noxious heat (45, 50, and 55°C) in *Trpv3*^*G*573*S*^ mutant in comparison to control. 45°C: (*n* = 7 for control, *n* = 13 for mutant), 50°C: (*n* = 10 for control, *n* = 16 for mutant), 55°C: (*n* = 11 for control, *n* = 17 for mutant). **(E)** Bar graph represents response score to acetone in *Trpv3*^*G*573*S*^ mutant and control littermates (*n* = 11 for control, *n* = 11 for mutant). **(F)** Bar graph represents latency to lick front paw when *Trpv3*^*G*573*S*^ mutants and control littermates were placed on a plate maintained at 0°C (*n* = 11 for control, *n* = 17 for mutant). NS, no significant difference, ^∗∗^*p* < 0.01, ^∗∗∗∗^*p* < 0.0001, Student’s unpaired *t*-test.

Next, we examined the role of TRPV3 in mediating noxious heat. We tested the response threshold by thermally stimulating the glabrous skin of the hind paw by radiant heat (Hargreaves Test). There was no difference in response latency between *Trpv3*^*G*573*S*^ mutant and control littermates in the Hargreaves test (Figure 4A). At higher temperatures, such as 46, 50, and 54°C, the latency of the hind paw lick was unchanged in the *Trpv3*^*G*573*S*^ mutant compared to control (Figures 4B–D). We also tested *Trpv3*^*G*573*S*^ mutants for acetone-induced cooling and 0°C-evoked cold responses. We observed that *Trpv3*^*G*573*S*^ mutants had increased latency to respond to acetone and 0°C cold plate (Figures 4E,F). To study the molecular mechanism of heat sensing in the skin, we stained TRPV1^+^ fibers in control and mutant mice. We found that TRPV1^+^ fibers were mainly located in the border regions between the epidermis and dermis (Supplementary Figure 1A). We detected rare TRPV1^+^ intraepidermal fibers (Supplementary Figure 1B), consistent with a previous study (Hsieh et al., 2012). Interestingly, there was no change in the innervation densities of TRPV1^+^ fibers in the *Trpv3*^*G*573*S*^ mutants when compared to control mice (Supplementary Figures 1C,D), which might be the reason why behavioral responses to noxious heat remain unaffected. Our results show that hyperactive TRPV3 channels did not alter the threshold or latency for sensing noxious heat. However, *Trpv3*^*G*573*S*^ mutants do show deficits in sensing cool and cold sensations.

### Evaluation of Itch Responses in *Trpv3*^*G*573*S*^ Mice

**FIGURE 5:**
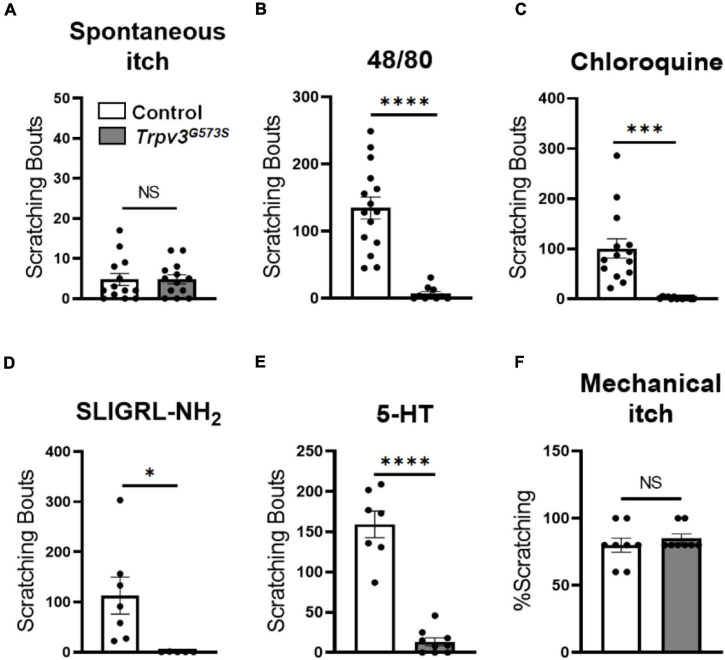
Assessment of itch sensation in *Trpv3*^*G*573*S*^ mutant mice. **(A)** Bar graph shows spontaneous scratching in *Trpv3*^*G*573*S*^ mutant and control littermates (*n* = 13 for control, *n* = 13 for mutant). **(B–E)** Bar graph showing scratching bouts in *Trpv3* G573S mutant in comparison to control in response to **(B)** compound 48/80 (*n* = 15 for control, *n* = 11 for mutant). **(C)** Chloroquine (*n* = 15 for control and *n* = 13 for mutant). **(D)** PAR2 agonist SLIGRL-NH2 (*n* = 7 for control and *n* = 5 for mutant) and **(E)** 5-HT (*n* = 7 for control and *n* = 7 for mutant). **(F)** Bar graph shows scratching responses to mechanical itch in *Trpv3*^*G*573*S*^ mutant and control littermates (*n* = 8 for each group). NS, no significant difference, ^∗^*p* < 0.05, ^∗∗∗^*p* < 0.001, ^∗∗∗∗^*p* < 0.0001, Student’s unpaired *t*-test.

A gain-of-function mutation in *Trpv3* is implicated in the development of dermatitis and spontaneous itch in conventional settings. However, in the absence of a specific pathogen, *S. aureus*, these mutant animals neither develop dermatitis nor show spontaneous scratching (Haraguchi et al., 1997; Asakawa et al., 2006; Imura et al., 2009). In our experiments, *Trpv3*^*G*573*S*^ mutants were raised in *S. aureus-free* environment, and we did not observe any dermatitis or spontaneous scratching in the *Trpv3*^*G*573*S*^ mutants (Figure 5A). To further examine the role of overactive TRPV3 channels in itch sensations, we tested a range of pruritogens-induced acute chemical itch behaviors in *Trpv3*^*G*573*S*^ mutation mice. Compound 48/80, chloroquine, PAR2 agonist SLIGRL-NH2, or Me-5-HT was injected subcutaneously in the nape of the animals, and scratching bouts were counted in control and *Trpv3*^*G*573*S*^ mutants. With the administration of different pruritogens, *Trpv3* mutants responded with dramatically reduced scratching bouts in comparison to control (Figures 5B–E). To test mechanical itch, a weak mechanical force was delivered on the shaved skin region behind the ear, and resultant scratching was counted. No significant difference was noted in *Trpv3* mutants in light touch-evoked pruritus when compared to control (Figure 5F). Hence, we found that the excessive activity of the TRPV3 channel is not sufficient to cause dermatitis, spontaneous scratching, or any difference in touch-evoked itch. However, it can result in a dramatic reduction in acute chemical itch. Our results suggest that overactive TRPV3 channels might not exert the primary role in itch transmission.

## Discussion

### Effect of Genetically Overactive TRPV3 Channels on Somatosensations and Epidermal Nerve Density

We observed that *Trpv3*^*G*573*S*^ mutant animals showed a deficit in sharp and punctate mechanical pain but no deficit in touch or sensory-motor coordination. In the thermosensation assays, we found that the hyperactivity of the TRPV3 channel did not change the latency to respond to radiant heat or the ability to sense intense noxious heat. Intriguingly, we observed a drastic degeneration of sensory nerve fibers in the epidermis in *Trpv3*^*G*573*S*^ mice. The observed attenuation in sensing cool, cold, acute mechanical pain and itch could be a result of decreased epidermal sensory innervations that mediates these modalities in naïve animals, whereas the sensory fibers involved in touch or heat could be the ones that remain functional in these mutants. Consistent with our behavioral readouts to noxious heat in *Trpv3*^*G*573*S*^, we did observe that innervation densities of TRPV1^+^ fibers in the border regions between epidermis and dermis remain unchanged in *Trpv3*^*G*573*S*^ animals in comparison to control mice.

A recent study shows that keratinocytes are involved in nerve fiber degeneration in small nerve neuropathy patients. In patients suffering from small fiber neuropathy, axon guidance cue netrin-1 is highly expressed in patients’ keratinocytes which can reduce sensory neurite outgrowth (Kress et al., 2021). Hence, the hypoinnervation could be an indirect effect of TRPV3 hyperactivity in non-neuronal skin cells such as keratinocytes that closely monitor or affect the epidermal sensory terminals. Hence, it would be interesting to examine whether hyperactive TRPV3 channels in keratinocytes can also deregulate the expression of axon guidance cues or other molecules that could affect the milieu of the epidermal nerve terminals to cause sensory fiber degeneration. Another possible mechanism for nerve degeneration could be that the hyperactivation of TRPV3 channels in the nerve terminal may prime the nerve terminal to release cues to cause nerve retraction or depletion. Additionally, *Trpv3*^*G*573*S*^ mutants also show drastically increased levels of CCL11 (∼250-fold) (Yoshioka et al., 2009), and elevated levels of this chemokine have been reported in many neurodegenerative disorders (Huber et al., 2018). Thus, upregulated cytokine release might also contribute to degeneration of nerve terminals in the epidermis.

Our results suggest that epidermal nerve degeneration in *Trpv3*^*G*573*S*^ mice might contribute to the deficits in acute itch transmission. However, epidermal innervation of nerve fibers is increased in the AEW-model of chronic itch conditions in control, but no such hyperinnervation is observed in *Trpv3^–/–^* upon AEW treatment (Yamamoto-Kasai et al., 2012). The authors did not find any difference in nerve fiber density in control and *Trpv3^–/–^* mice under normal conditions (Yamamoto-Kasai et al., 2012). In chronic itch induced by AEW, various pathways come into action that precipitates to induce hyperinnervation of the epidermis and might require the participation of TRPV3 channels to induce nerve sprouting in the epidermis in chronic itch conditions. How activation of TRPV3 channels controls the growth of epidermal nerve terminals in control, and chronic itch conditions is a question that remains to be answered.

### Role of Hyperactive TRPV3 Channels in Itch and Olmsted Syndrome

In the acute itch assays, we observed a decrease in scratching responses to various pruritogens in the *Trpv3*^*G*573*S*^ mutant in comparison to the control. Gain-of-function mutations in the *Trpv3* gene, including the G573S point mutation, cause a form of palmoplantar keratoderma known as Olmsted syndrome, an autosomal dominant genodermatoses phenotypically characterized by palmoplantar keratoderma and periorificial keratotic plaques. In addition to these symptoms, not all, but some cases of Olmsted syndrome exhibit lesion-associated pain, and or itch, with varying degrees of intensity (Poulin et al., 1984; Lucker and Steijlen, 1994; Nofal et al., 2010; Lai-Cheong et al., 2012). However, only ∼22% of patients suffering from Olmsted syndrome suffer from pruritus, and ∼52% of patients suffer from pain (Mevorah et al., 2005; Tao et al., 2008). Hence a direct link between a gain-of-function mutation in *Trpv3* and itch cannot be established.

The environmental pathogens and the background of the animal can play a very crucial role in constructing a unique immune profile that dictates the somatosensory responses, including itch. The notion that G573S gain-of-function mutation in *Trpv3* does not unswervingly result in pruritus is also supported by the findings where DS-*Nh* animals harboring a spontaneous *Trpv3*^*G*573*S*^ mutation when raised in the *S. aureus*-free environment do not develop itch and/or dermatitis (Haraguchi et al., 1997; Asakawa et al., 2006; Imura et al., 2009). Consistently, we observed that *Trpv3*^*G*573*S*^ mice housed in the specific-pathogen free (SPF) conditions did not show spontaneous scratching behavior or develop any dermatitis at any time during their lifetime of a year (data not shown). Interestingly, when *Nh* mutant is introduced in a different background such as NC/Nga, animals harboring gain-of-function mutation in the *Trpv3* gene do not develop any scratching or dermatitis even in the presence of *S. aureus* (Imura et al., 2009). Under conventional conditions, DS-*Trpv3*^*G*573*S*^ mutant animals exhibit an increased infiltration of mast cells in the skin, increased levels of IL2, IL-13, IL-17, eotaxin, G-CSF, GM-CSF, IFN-y, MCP-I, MIP-1α, IL-1α, IL-6, IL-9, IL-10, and IL-18 in comparison to *Trpv3*^G573S^ mutant animals raised in the SPF conditions (Imura et al., 2009). Contrastingly, Yoshioka et al. (2009) found that a transgenic *Trpv3*^*G*573*S*^ mutant on either C57BL or DS background shows increased scratching, but whether the mutants are raised in the SPF or conventional conditions is not specified. Additionally, TRPV3 antagonists such as forsythoside B and citrusinine-II are shown to attenuate acute and chronic itch and pain; however, these compounds have known (Jiang et al., 2012; Liu et al., 2019), or could have unknown off-targets that might result in suppression of itch (Zhang et al., 2019; Han et al., 2021). Also, no conclusive evidence is available that can indicate how an overactive TRPV3 channel can collaborate with genetic, environmental, and/or inflammatory triggers to induce itch in the patients harboring gain-of-function mutations in the *Trpv3* gene. Additionally, patients suffering from Olmsted syndrome display genetic heterogeneity where different naturally occurring mutations in the *TRPV3* gene (Wilson et al., 2015) show similar phenotypes along with clinical heterogeneity. The correlation between allelic and phenotypic heterogeneity in Olmsted syndrome is yet to be explored.

Patients suffering from Olmstead syndrome also show diversity in the severity of alopecia. Some human patients do show alopecia totalis, others show patches of hair loss, hypotrichosis, and/or brittle hair quality, and in rare cases, patients have normal hair (Lin et al., 2012; Wilson et al., 2015; Ni et al., 2016). TRPV3 is shown to control hair development both in humans and rodents. Agonist-mediated activation of TRPV3 *in vitro* by eugenol or 2-APB results in dose-dependent inhibition of human hair shaft elongation and facilitates the premature entry into catagen stage (Borbiro et al., 2011). In rodents, a hyperactive TRPV3 channel can induce an inefficient differentiation of follicular keratinocytes (Song et al., 2021), and the anaphase is recorded to be more persistent, whereas the telophase is absent in DS-*Nh* mice at P21 (Imura et al., 2007). In our mouse model, *Trpv3*^*G*573*S*^ mutants did have brittle hair that had drastically reduced hair diameter and length and were not completely hairless (Figure 1A).

In relation to skin thickening due to gain-of-function mutation, while Olmsted patients show diffuse palmoplantar keratoderma, which is an abnormal thickening of the skin, and *Trpv3*^*G*573*S*^ mutant mice also showed thickened skin. Ambient activation of TRPV3 can result in increased proliferation in keratinocytes in mice (Wang et al., 2021).

Thus, diverse phenotypic spectrum observed in humans and mice due to hyperactive TRPV3 channels observed could be attributable to species-specific differences, allelic or non-allelic genetic diversity, and/or environmental triggers, which could strongly influence the immune responses resulting in inter-and intra-species differences and the precise mechanism of phenotypic diversity in hair and skin between mice and humans is yet to be explored.

## Conclusion

In this work, we observe that hyperactivation of TRPV3 channels results in attenuation of somatosensory responses to cool and cold, sharp and punctate mechanical pain, and acute chemical itch, which could be a result of depletion of sensory innervation in the epidermis. Importantly, we infer that the hyperactivity of TRPV3 channels is not sufficient to induce itch and hence is not a suitable target to design drug interventions to treat itch.

## Data Availability Statement

The raw data supporting the conclusions of this article will be made available by the authors, without undue reservation, to any qualified researcher.

## Ethics Statement

The animal study was reviewed and approved by the Institutional Animal Care and Use Committee, University of Michigan.

## Author Contributions

BD conceptualized the study. MF and AS performed histology studies. MF, HS, LH, JL, DM, and TV performed behavioral tests. BD, MF, HS, LH, and TV analyzed the data. HX provided *Trpv3*^*G*573*S*^ mice. BD and MF wrote the manuscript. All authors contributed to the article and approved the submitted version.

## Conflict of Interest

The authors declare that the research was conducted in the absence of any commercial or financial relationships that could be construed as a potential conflict of interest.

## Publisher’s Note

All claims expressed in this article are solely those of the authors and do not necessarily represent those of their affiliated organizations, or those of the publisher, the editors and the reviewers. Any product that may be evaluated in this article, or claim that may be made by its manufacturer, is not guaranteed or endorsed by the publisher.

## Supplementary Material

Supplementary Figure 1 Immunohistochemical chemical detection of TRPV1 in the glabrous skin. **(A)** Touchdown skin area of glabrous skin sections from mutant and control were immunohistochemically probed for TRPV1 receptor. Left panel shows images from the touch-down area (scale 50 μm). **(B)** Images show immunostained TRPV1^+^ fibers in the epidermis of control and mutants (scale 20 μm). White dashed line in the images indicate the extent of epidermis. **(C)** Schematic showing innervation pattern of TRPV1 in the footpad skin. Region of interest (ROI), represented by dashed rectangle, was randomly selected in the dermis to sample the fluorescence density of TRPV1-positive nerve fibers. **(D)** Graph bar shows quantified fluorescence intensities in control and mutants (*n* = 7–8 sections from 2 mice for each group).
