# Supplementary figures and images for: Abnormal Somatosensory Behaviors Associated With a Gain-of-Function Mutation in TRPV3 Channels
Source: Front Mol Neurosci. 2022 Jan 4;14:790435. doi: 10.3389/fnmol.2021.790435 (PMC8764439; doi:10.3389/fnmol.2021.790435)

Figure S1

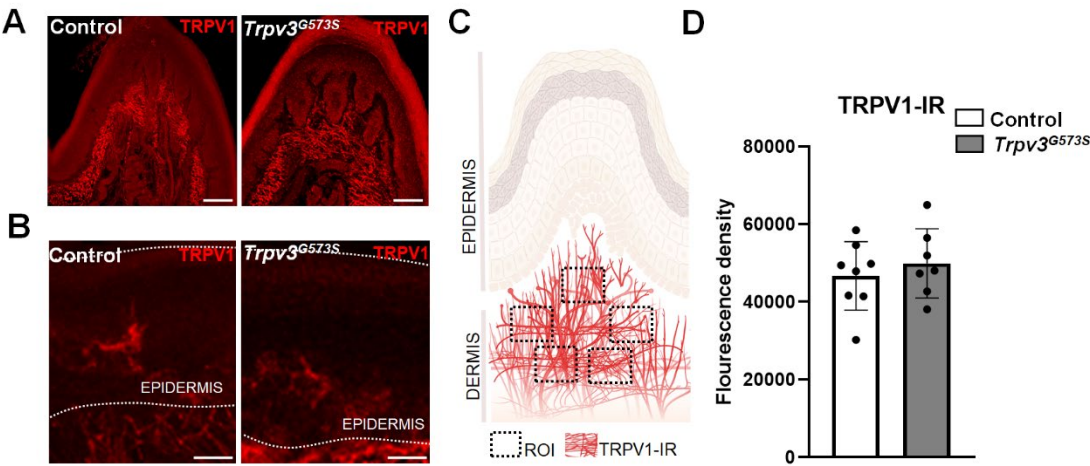

Supplement: Supplementary Figure 1 — Immunohistochemical chemical detection of TRPV1 in the glabrous skin. (A) Touchdown skin area of glabrous skin sections from mutant and control were immunohistochemically probed for TRPV1 receptor. Left panel shows images from the touch-down area (scale 50 μm). (B) Images show immunostained TRPV1+ fibers in the epidermis of control and mutants (scale 20 μm). White dashed line in the images indicate the extent of epidermis. (C) Schematic showing innervation pattern of TRPV1 in the footpad skin. Region of interest (ROI), represented by dashed rectangle, was randomly selected in the dermis to sample the fluorescence density of TRPV1-positive nerve fibers. (D) Graph bar shows quantified fluorescence intensities in control and mutants (n = 7–8 sections from 2 mice for each group). [file Image_1.pdf]
